# Effects of COVID-19 related economic threat on political conservatism, xenophobia, and racial bias in the United States

**DOI:** 10.1371/journal.pone.0309766

**Published:** 2024-09-18

**Authors:** Crystal X. Wang, Stanley J. Huey, Miriam P. Rubenson

**Affiliations:** 1 Department of Psychiatry, HIV Neurobehavioral Research Program, University of California, San Diego, San Diego, California, United States of America; 2 Department of Psychology, University of Southern California, Los Angeles, California, United States of America; 3 Seattle Children’s Hospital, Seattle, Washington, United States of America; National Taiwan University, TAIWAN

## Abstract

The uncertainty-threat model of conservatism posits that people turn to political conservatism to protect themselves from perceived threats; indeed, studies show increases in conservative ideology and outgroup bias following threat priming. The COVID-19 pandemic is an unprecedented threat that has had devastating effects on the health and economic lives of Americans. Concerns surrounding the threat of COVID-19 may have secondary effects on other aspects of American life, such as political and anti-Asian racial bias. The current studies explored the effects of COVID-19 related threats on expressed political conservatism, xenophobia, and racial bias under the uncertainty-threat model. Study 1 assessed the effects of priming health or economic risks of COVID-19 (vs. control), and found that economic threat led to increased xenophobia, but had no effects on overall conservatism. Study 2 then investigated whether the effects of COVID-related economic threat prime extended to racial bias, and explored moderators and mediators of effects. Results showed that the economic threat prime increased perceived group-status threat, and indirectly increased conservatism, xenophobia, and racial bias through the mechanism of perceived group-status threat. Effects were greatest for those impacted financially by the pandemic. In general, our studies provide support for the uncertainty-threat model with the novel threat of the COVID-19 pandemic. Implications for understanding potential shifts in conservatism and bias in response to future threats in the United States are discussed.

## Introduction

The uncertainty-threat model of conservatism posits that political conservatism functions to alleviate anxiety from uncertainty and perceived threat [1]. In general, conservative ideologies oppose change and support the status quo, whereas liberal ideologies advocate for change and equality through disruption of social hierarchies [1, 2]. Thus, conservative belief systems serve to reduce ambiguities and relieve anxiety [1–3]. Political conservatism has been linked to increased threat sensitivity, need for order or structure, and ambiguity intolerance in numerous studies [1, 4] In support of the uncertainty-threat model of conservatism, past studies find increased endorsement of conservatism and Republican party affiliation after major national threats in the United States such as the terrorist attacks of September 11^th^, 2001, and the Great Recession of 2007–2009 [5, 6].

Although conservatism can be a stable and enduring trait throughout an individual’s lifetime, the presence of threatening information can increase endorsement of conservative ideology, even among those who are otherwise low in conservatism [7–10]. For example, Nail and McGregor showed that threat can cause those who identify as liberal to endorse traditionally conservative ideology to the same degree as conservatives [8]. Specifically, they found that exposure to mortality and injustice threats caused increased endorsement of conservative stances on policies such as capital punishment, abortion, and gay rights among conservative *and* liberal participants [8].

Besides political attitudes, threat may also increase xenophobia or racism. In addition to correlational studies showing associations between threat and bias [11], experimental studies showed that threat primes increased xenophobia and bias. Utilizing the uncertainty-threat model framework, researchers have found relationships between political affiliation and outgroup bias through the mechanism of threat and uncertainty [4]. Specifically, they found that conservatives with higher uncertainty-avoidance and who perceived greater threat from immigrants were more likely to endorse xenophobic attitudes [4]. Additionally, in a series of experiments, Craig and Richeson [7, 12, 13] found that presenting threatening news about the rapid increase of the Hispanic population in the United States (i.e., the “majority-minority phenomenon”) led to greater perceived threat and subsequent conservative shifts and increased anti-immigrant attitudes among participants of diverse racial/ethnic backgrounds. Similarly, terror management studies show that reminders of one’s own mortality can experimentally prime ingroup bias and negative attitudes towards other ethnic groups [14–16]. Although there is extensive research demonstrating the priming effects of various threats on political and racial attitudes, it remains unclear how the uncertainty-threat model applies to the relatively novel threat of COVID-19, which has been considered the greatest international crisis in the 21^st^ century [17, 18].

COVID-19 was first identified in Wuhan, China in December 2019, with confirmed spread to the United States by February 2020. Since then, the COVID-19 pandemic has created widespread devastation throughout the United States–over 103 million cases and 1 million deaths as of March 2023 [19]. The pandemic has impacted almost every aspect of American life, with significant health, economic, and social repercussions. Unsurprisingly, studies find increased levels of anxiety and depression in response to COVID-19 that far surpass normative estimates [20–22]. It is unclear, however, whether high levels of distress and uncertainty during this pandemic influenced opinions or attitudes about politics and race, although correlational data show increases in racial bias and xenophobia, specifically towards Chinese people since early 2020 [23–25]. During this time period, American politicians frequently referred to COVID-19 as the “Chinese virus,” with then-President Trump publicly using this expression more than 20 times in March 2020 alone, and repeatedly claiming that the virus was “China’s fault” [26]. Associating COVID-19 with China and the Chinese has been linked to surges in anti-Chinese sentiment across the country; since COVID-19 was first reported in the news, there have been numerous reports of physical assaults on Asians by assailants who blame them for COVID-19 [27, 28]. The estimated increase in anti-Asian hate crimes at the start of the pandemic was well over 300% [28]. Moreover, a text-based analysis of internet trends in 2020 found increases in Sinophobia, including racist slurs and threats, since the start of the pandemic, and blame towards Chinese people for the spread of COVID-19 [29]. This increase in Sinophobia may be associated with concurrent political events. For example, Hswen and colleagues found a significant increase in anti-Asian attitudes immediately after President Trump tweeted the term “Chinese Virus” on March 16, 2020 [30]. Although anti-Asian sentiment was strongest in the months following the outbreak, the surge in hate crimes persisted well into the pandemic, culminating in several mass shootings of Asian Americans within the last few years [31, 32].

Furthermore, there may have been political divisions in reactions towards China in relation to COVID-19, with Republican lawmakers favoring harsher measures than Democrats. Republican politicians have been outspoken in blaming China for the pandemic, and a detailed memo written by the National Republican Senatorial Committee in April 2020 advised an aggressive attack on China for the COVID-19 pandemic [33, 34]. These attitudes were echoed by the general public, wherein those who identified as Republican were far more likely to endorse anti-China sentiment than Democrats in polls conducted in mid-2020 [35]. In addition, Republicans tended to demonstrate greater anti-Asian sentiment and were less supportive of Asian American hate crime victims. For example, Americans who identified as Republican were significantly more likely to report anti-Asian bias than Democrats or Independents [36], and of the representatives who voted against the COVID-19 Hate Crimes Act, which supported the predominantly Asian victims of hate crimes, all 62 were Republican [37].

According to the uncertainty-threat model of conservatism, the COVID-19 pandemic may cause surges in conservatism across the country. For example, some conservative policies, such as restricting immigration and travel, may help reduce anxieties related to contagion. However, other conservative policies such cutting government spending on welfare benefits during the economically stressful COVID-19 period may actually increase anxieties related to financial burden. In addition, explicitly associating COVID-19 with the Chinese may increase xenophobic or racist attitudes. Thus, the present project investigates the effects of various COVID-19 related threats on the endorsement of conservative policies, xenophobia, and racial bias across two studies. In Study 1, we tested the effects of different forms of COVID-19 related threat on conservatism and xenophobia. In Study 2, we tested whether the effects of COVID-19 related threat extend to racial attitudes, and explored potential mechanisms of effects (e.g., perceived threat) found in past literature [4, 12]. Based on the uncertainty-threat model and prior work by Craig and Richeson [7, 12, 13], we predicted that COVID-related threat would prime feelings of anxiety and uncertainty, which would be ameliorated through greater endorsement of conservatism, xenophobia, and racial bias. Our results can provide insight on understanding the shifts in political and xenophobic or racial attitudes across the United States during the era of COVID-19 under the uncertainty-threat model, and have implications for helping predict attitudinal shifts during future large-scale threats.

## Study 1

We first sought to investigate which COVID-19 related threats, if any, affect political and xenophobic attitudes. Media coverage in the early pandemic tended to revolve around two COVID-19 related concerns: health risks associated with the virus, and the dramatic effects on the economy [38–40]. Thus, Study 1 sought to test the effects of these two COVID-19 related threats, health and economic (vs. control), on political ideology and xenophobia. Prior studies showed that perceptions of COVID-19 threats were associated with high rates of anxiety and uncertainty intolerance [41, 42]. According to the uncertainty-threat model, conservatism operates to reduce feelings of anxiety and uncertainty from perceived threats [1]; thus, we argue that both our health and economic threat conditions should lead to greater endorsement of conservatism. In addition, participants in both threat conditions should endorse more xenophobic attitudes than control.

However, COVID-19 related economic threat may lead to stronger effects than health threat. During the time this study was conducted (April 30-May 1, 2020), the economic consequences of COVID-19 (e.g., 23% unemployment rate; 26.5 million newly unemployed) affected more Americans than health-related consequences (e.g., 928,619 contracted the virus, 52,000 dead from COVID-related causes; CDC 2020). In addition, conservatives tended to be more concerned with the economic repercussions of COVID-19, and less concerned about health risks [43]. For example, a poll conducted between May 5 and May 10, 2020 showed that 61% of Republicans favored the re-opening of non-essential businesses, versus only 29% of Democrats [44]. Therefore, the present study compares both forms of threat to each other and control.

### Method

#### Participants

One hundred and eighty-one participants were recruited from Prolific, an online research participant pool, and paid $0.80 for their participation in the survey that took an average of 5 minutes to complete. Participants’ mean age was 31.72 years, and they were mostly male (62.2%), and White (67.8%), with the remaining participants identifying as Black (8.9%), Latino (8.3%), Asian (11.7%) or Other (3.3%). Approximately 42.2% identified as Democrat, 26.7% as Republican, 21.7% as Independent, 7.2% as other, and 7.2% as no preference. A prior power analysis conducted with the program G*Power 3 [45], with the alpha (Type I error) set to .05, and power set to 80%, found that a sample size of 158 was needed to detect a medium effect size of η_*p*_^2^ = 0.06.

#### Procedure

The study was conducted between April 30 to May 1, 2020. Human subjects approval of this study was obtained from the University of Southern California Institutional Review Board. Written informed consent was obtained from all participants prior to engaging in the study. Upon approval, participants were randomly assigned to read one of three articles presenting information on COVID-19 or an unrelated control topic (S1 Appendix). The articles were modeled on stimuli from Craig and Richeson’s threat-priming study [7] and included one of three threat scenarios: (1) surges in cases and deaths from COVID-19 (health threat condition), (2) surges in unemployment rates from COVID-19 (economic threat condition), or (3) surges in social media use over the past 15 years (no threat control). The health threat condition reported data from the Centers for Disease Control and Prevention showing the exponential surges in infection and mortality rates from COVID-19 [19]. The economic threat condition reported on the exponential surge in unemployment, based on reports from the U.S. Bureau of Labor Statistics [46]. Finally, the control condition reported on the exponential surge in social media usage since 2005, based on reports from the Pew Research Center. All articles were similar in length (range: 182–200 words), and content (e.g., similar word choice, reporting of statistics, and use of graphics).

Next, participants answered questions assessing their endorsement of several political policies, attitudes towards foreigners, and relevant demographics. At the end of the survey, participants were asked which article they read as an attention check. Out of the 181 participants, 180 passed the attention check and were included in subsequent analyses. All data was de-identified following data collection.

#### Measures

*Political stances*. Questions assessing endorsement of liberal or conservative policies were borrowed from a prior study investigating the effects of race-based threat on conservatism [7]. Sample questions included: “Please state how strongly you support or oppose same-sex marriage” and “Please state how strongly you are in favor of increasing or decreasing the minimum wage” on a scale from 1 (i.e., “strongly oppose”) to 7 (i.e., “strongly support”). We reverse-coded liberal items, and summed and averaged all 13 items for a composite conservatism score, with higher scores indicating greater conservatism. Reliability for the conservatism scale was good (*α* = .89).

Participants were also asked 5 author-constructed questions assessing how much they endorsed policies specific to COVID-19. These policies included: (1) mandatory wearing of masks, (2) extending shelter-in-place restrictions, (3) reopening public parks, (4) free, federally funded COVID-19 testing, and (5) free, federally funded treatment for COVID-19. A sample question asked, “In response to the current COVID-19 pandemic specifically, please state how strongly you support or oppose mandatory wearing of masks in public” on a 7-point scale from 1 (i.e., “strongly oppose”) to 7 (i.e., “strongly support”). The items were chosen based on common COVID-19 related policies reported in early 2020 [47]. Items 1, 2, 4, and 5 were policies commonly endorsed by liberals, and item 3 by conservatives. We reverse-coded liberal items, and averaged all items for a composite score of COVID-19 specific policies, with higher scores indicating greater conservatism. The reliability for COVID-19 specific questions was good (*α* = 0.82).

*Xenophobia*. To measure xenophobia, we asked five questions assessing attitudes towards immigration [7], and four questions assessing attitudes towards travel restrictions (created for this study). The five immigration items (e.g., “Please state how strongly you are in favor of increasing or decreasing immigration to the US”) had good reliability (*α* = 0.78). Scores were totaled to create a composite anti-immigration variable. The travel restrictions items asked participants whether they favored restricting travel for the following groups from areas with highly publicized outbreaks of COVID-19 at the time of data collection: (1) Chinese nationals, (2) Italian nationals, (3) South Korean nationals, and (4) New Yorkers (e.g., “Please state how strongly you support or oppose restricting travel from Chinese nationals into the US”), and had excellent reliability (*α* = 0.94). Although our exploratory analyses compared the endorsement of travel restrictions against the Chinese, Italians, South Koreans, and New Yorkers to one another, endorsement of travel restriction against South Korean nationals in particular was our main outcome of interest to measure xenophobia, as South Korea was an East Asian country proximal to China, but with low rates of COVID in 2020 [48]. Given their well-controlled COVID during that time period [48], greater endorsement of travel restrictions against South Koreans following the manipulation suggested xenophobic or anti-Asian attitudes. China was included due to its role as the epicenter of the COVID-19 pandemic, and Italy and New York were included as controls due to their extremely high rates of COVID during that same time period [49, 50]. All items for the two measures were assessed on a 7-point scale with responses ranging from 1 (i.e., “strongly oppose”) to 7 (i.e., “strongly support”). Higher scores for both the anti-immigration and travel restriction item regarding South Korea represented greater xenophobia.

### Results

#### Statistical analyses

For preliminary analyses, a series of one-way analysis of variance (ANOVA) tests were conducted to assess for differences between conditions on participant demographics. Independent samples t-tests then assessed for differences in outcome (e.g., conservatism, xenophobia) between Republicans and Democrats. Exploratory analyses also assessed whether travel restrictions were more likely to be endorsed for some nationalities compared to others. Bonferroni adjustments were made for multiple comparisons.

As for primary analyses, a series of analysis of covariance (ANCOVA) models were conducted to examine experimental condition effects on outcomes (e.g., conservatism, xenophobia). Given evidence for political divides by gender and age [51], these demographic variables were entered into all models as covariates. For significant findings, Least Significant Difference (LSD) post-hoc tests were conducted to disaggregate condition effects.

#### Preliminary analyses

There were no significant differences between experimental groups by age, gender, ethnicity, or political affiliation, *p* > .05, suggesting successful randomization. Republicans were significantly higher in political conservatism, *t*(123) = 10.35, *p* < .01, and more in favor of anti-immigrant policies, *t*(123) = 7.18, *p* < .01, and overall travel restrictions, *t*(123) = 3.50, *p* < .01, than Democrats. Similarly, Republicans were more likely to endorse conservative stances specific to COVID-19 (e.g., temporarily pausing immigration) than Democrats, *t*(123) = 4.41, *p* < .01.

**Fig 1:**
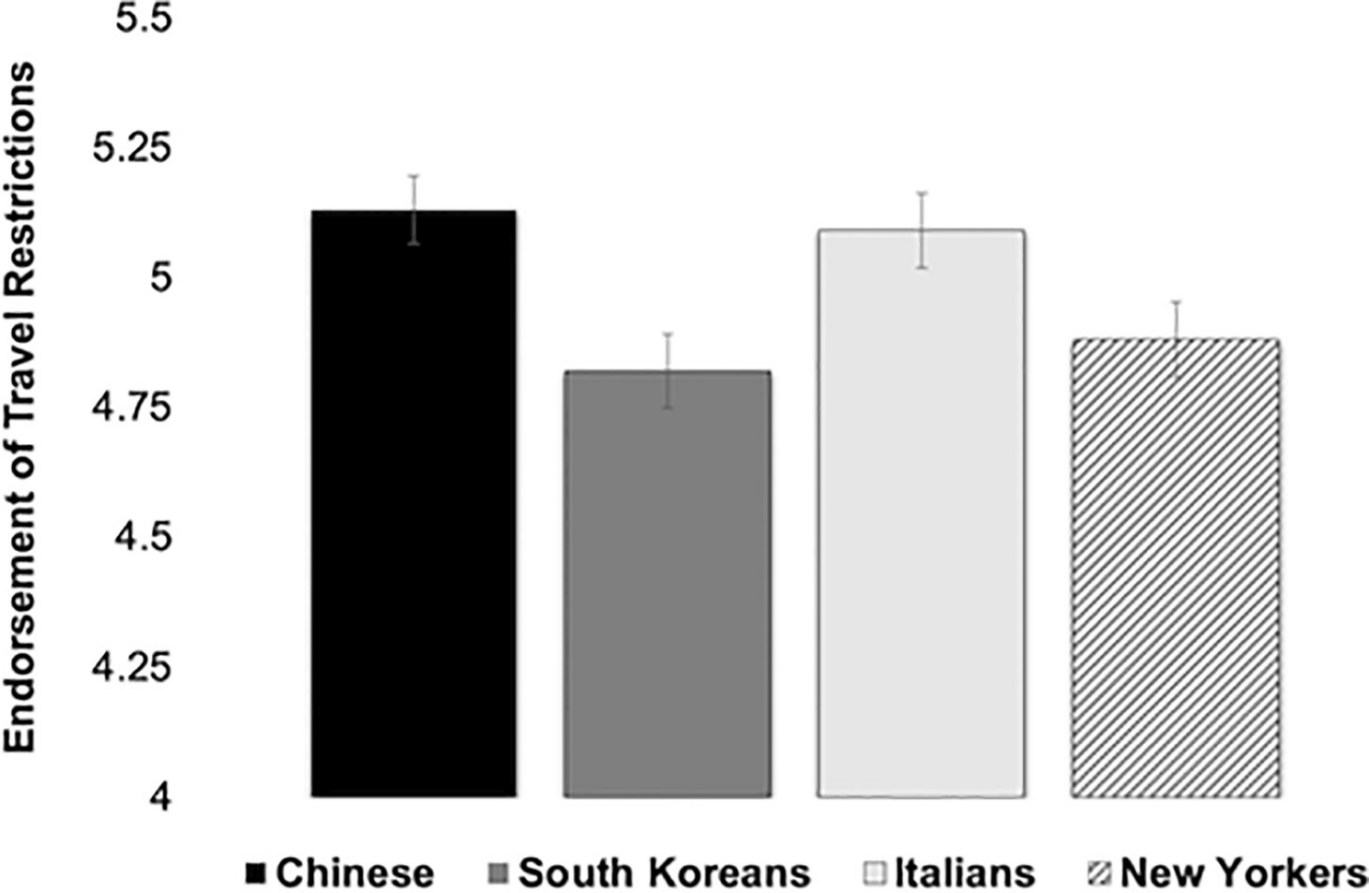
Endorsement of travel restrictions by country in Study 1.

Across participants, there was greater endorsement of travel restrictions against Chinese (*M* = 5.13, *SD* = 1.75) than South Koreans (*M* = 4.82, *SE* = 1.91), *t*(179) = 4.85, *p* < .01. In addition, there was greater endorsement of travel restrictions against Italians (*M* = 5.09, *SE* = 1.72) than South Koreans, *t*(179) = 4.67, *p* < .01. There was no difference in endorsement of travel restrictions for the Chinese vs. Italians or New Yorkers, Italians vs. New Yorkers, or South Koreans vs. New Yorkers, *p* > .05 (Fig 1).

#### Primary analyses

We assessed condition effects on policy endorsement using ANCOVA tests. Contrary to expectation, no significant differences were found between economic threat (*M* = 59.84, *SD* = 15.01), health threat (*M* = 59.47, *SD* = 14.85), and control (*M* = 61.37, *SD* = 14.89) on political conservatism, *F*(2, 179) = .33, *p* = .72, η_*p*_^2^ < .01. In addition, no condition effects were found for COVID-specific political policies, *F*(2, 179) = .67, *p* = .51, η_*p*_^2^ < .01.

**Fig 2:**
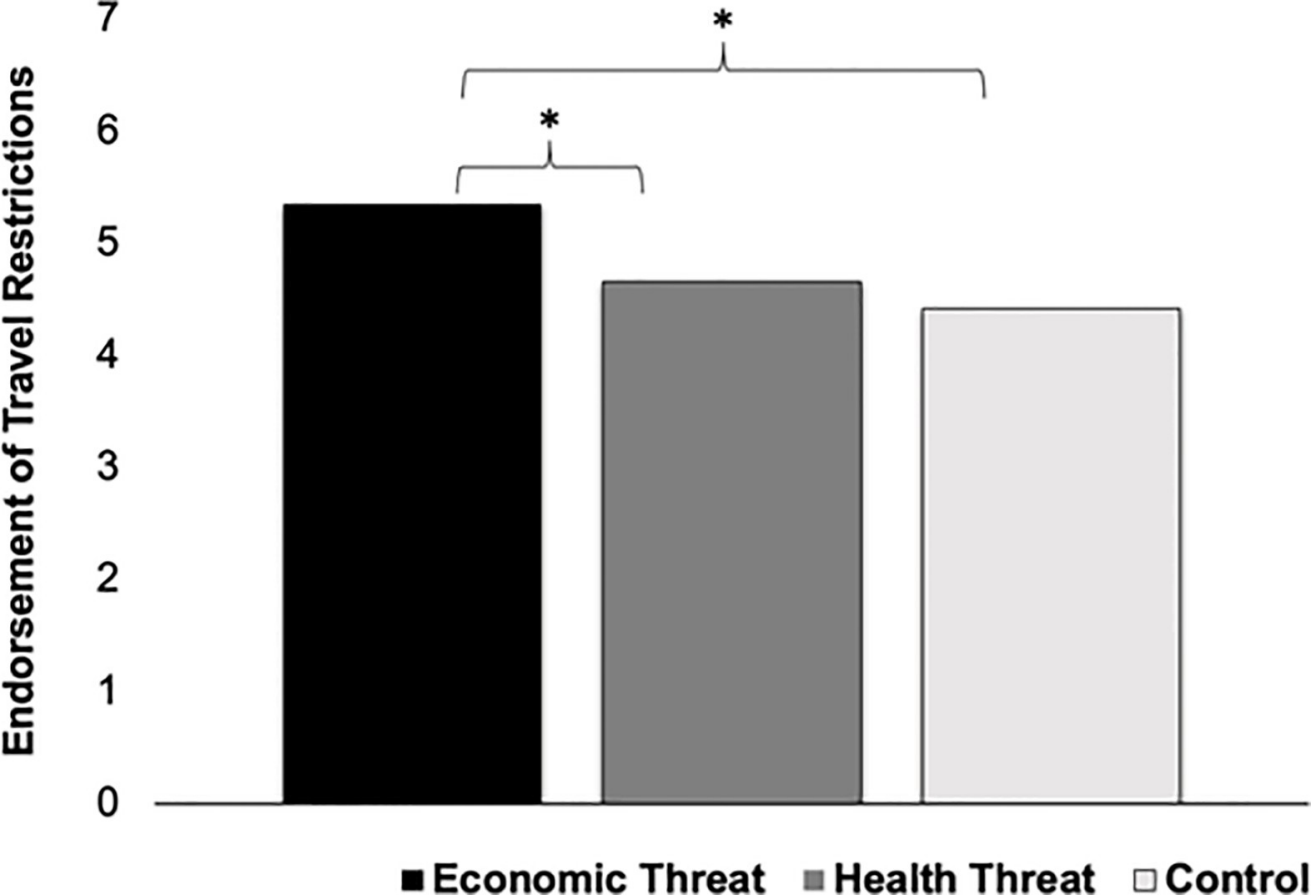
Condition effects on endorsement of travel restrictions against South Korean nationals in Study 1.

Condition effects were found for one measure of xenophobia, *F*(2, 179) = 4.03, *p* = .02, η_*p*_^2^ = .04 (Fig 2). LSD tests revealed that economic threat (*M* = 5.35, *SD* = 1.90) led to significantly greater endorsement of travel restrictions against South Korean nationals than health threat (*M* = 4.66, *SD* = 1.71), *p* = .04, and control (*M* = 4.42, *SD* = 2.02), *p* < .01. There was no significant difference between the health threat and control condition, *p* = .49. Follow-up analyses assessed for condition effects on composite travel restrictions against all 4 groups, and travel restrictions against each group separately. Similar to the results for South Korean nationals, there were condition effects for the composite of all 4 groups, *F*(2, 179) = 3.52, *p* = .03, η_*p*_^2^ = .04, Chinese nationals, *F*(2, 179) = 4.83, *p* < .01, η_*p*_^2^ = .05, and Italian nationals, *F*(2, 179) = 4.07, *p* = .02, η_*p*_^2^ = .04, such that economic threat led to significantly greater endorsement of travel restrictions than health threat and control, with no significant differences between health threat and control. No condition effects emerged for travel restrictions against New Yorkers, *F*(2, 179) = .63, *p* = .53, η_*p*_^2^ = .01, or attitudes towards immigration, *F*(2, 179) = 1.29, *p* = .28, η_*p*_^2^ = .02.

### Discussion

Results from Study 1 partially confirm our hypotheses. As expected, we found that priming COVID-19 related economic threat led to greater xenophobia than the health prime or control. Specifically, economic threat (vs. health threat or control) led to greater endorsement of travel restrictions against the Chinese, South Koreans, and Italians, but not New Yorkers, despite their extremely high levels of COVID-19 in 2020 [49]. Of particular interest is the endorsement of travel restrictions against South Korean nationals, given South Korea’s well-controlled COVID-19, which garnered considerable media attention during that time [48]. Thus, restricting travel against South Korean nationals likely reflects xenophobia due to anti-Asian bias rather than any justified reason for social distancing. However, it is also possible that participant endorsement of travel restrictions against any foreign nationals and pausing immigration reflected desires to prevent the spread of COVID-19, rather than racism per se [52]. Although xenophobia is often linked with racism and outgroup bias, with some researchers considering it a form of culturally-based racism [53], there is a clear difference in the utility of xenophobia vs. racism. Unlike temporary immigration and travel restrictions, racist attitudes cannot plausibly prevent the spread of COVID-19.

Contrary to expectations, we found no priming effects of economic threat on political conservatism. However, some studies have found that threat only primed conservatism through certain threat-based mechanisms, such as perceived group-status threat [12, 54], with no direct effects on conservatism. In addition, it is possible that threat only affected political or xenophobic/racial attitudes among those who were directly impacted by COVID-19. As a result, Study 2 expands on the findings in Study 1 by investigating whether COVID-19 threat also affects racial attitudes, and tests mediators and moderators of effects.

## Study 2

Study 1 showed that COVID-related economic threat, but not health threat, had effects on xenophobia. As predicted, the economic prime led to greater xenophobia than health threat or control; however, it is unclear whether these effects extend to racial bias. Also, Study 1 did not explore potential mechanisms to explain our condition effects, and we were unable to assess for moderators of effects, such as political orientation, due to the limited number of Republicans in the sample. Study 2 investigates in greater depth the effects of economic threat on xenophobia and racial bias with a larger, more politically balanced sample, and additional questions to assess for potential mechanisms.

To clarify results from Study 1, we removed the health threat condition and instead included a non-threatening COVID-19 prime condition. This non-threat condition describes surges in the use of Zoom and other video conferencing platforms since January 2020 (S2 Appendix). If significant differences between the economic threat vs. non-threat and control conditions emerge, differences may be due to the economic threat itself and not just the priming of COVID-19.

We predicted that economic threat would lead to greater conservatism, xenophobia, and racial bias than the other two conditions. To assess for potential moderators of priming effects, we increased our sample size, collected a roughly equal number of Republicans, Democrats, and Independents, and assessed for COVID-related financial impact. Given prior literature demonstrating equivalent effects of threat priming for both liberals and conservatives, we expected similar results. However, due to the dramatic political divide in responses to the COVID-19 pandemic, we ran exploratory analyses assessing for any interactions between condition and political affiliation. We also predicted that COVID-related financial impact would moderate effects, such that those who lost jobs or income due to COVID-19 would also be more sensitive to priming effects.

Finally, we predicted that perceived group-status threat, or perceptions of threat to American social structures, would mediate the effects of the economic prime on outcome. Craig and Richeson found that experimentally priming the “majority-minority” phenomenon increased conservatism through the mechanism of perceived group-status threat [12]. Although we employed a subtler prime that does not mention race or ethnicity, we expected similar results, such that perceived group-status threat would mediate threat priming effects on conservatism and bias. Consistent with findings of prior experimental threat priming studies, we made these specific hypotheses: (1) the economic threat prime (vs. non-threat and control) will be associated with greater perceived group-status threat (path *a*) [55], (2) perceived group-status threat will be associated with increases in conservatism, xenophobia, and racial bias (path *b*) [54], and (3) economic threat will be associated with greater conservatism, xenophobia, and racial bias through the mechanism of group-status threat (path *ab*) [12].

### Method

#### Participants

Two hundred and fifty-two participants were recruited from Prolific and compensated $1.43 for Study 2. We used stratified random sampling to recruit roughly equal-sized groups of Republicans (30.6%), Democrats (34.1%), and Independents (30.2%), with the remainder identifying as other (3.6%) or no preference (1.6%). Participants were predominantly White (76.6% White, 12.3% Asian, 7.1% Black, and 7.5% Latino), and mostly male (54.4%); the mean age was 32.26 years, and 38.5% had lost their job or a significant source of income due to COVID-19. A priori power analysis was conducted with the program G*Power 3 [43], with power set to 80%, and alpha set to 0.05, found that a sample size of 196 was needed to detect a medium effect size of η_*p*_^2^ = 0.06.

#### Procedure

Study 2 was conducted on May 4, 2020. Human subjects approval of this study was obtained from the University of Southern California Institutional Review Board. Written informed consent was obtained from all participants prior to study involvement. Similar to Study 1, participants were randomly assigned to read one of three articles. The economic threat and control articles were the same as those used in Study 1, but the new non-threatening COVID-19 article was created for this study. This non-threat condition described surges in video conferencing use during the pandemic, specifically with the online meeting platform Zoom, and omitted explicit references to threatening information. The article was similar to the others in terms of length, language, and structure (S2 Appendix). After reading their assigned article, participants answered questions assessing their endorsement of several political policies, xenophobic and racial attitudes, and relevant demographic information. All 252 participants passed the attention check, which was identical to that used in Study 1. Participant data was de-identified following data collection.

#### Measures

*Political stances*. Measures of political ideology were the same as those used in Study 1. The 13-item conservatism scale had good reliability (*α* = .89). We added two new items to the 5-item scale assessing attitudes towards policies specific to COVID-19. These questions assessed participants’ attitudes towards re-opening non-essential businesses, and having China pay reparations for the pandemic—a stance that had been frequently endorsed by the Republican party [34]. Reliability for the updated 7-item COVID-19 scale was good (*α* = 0.80).

*Xenophobia*. To assess xenophobia, we used the same questions assessing attitudes towards immigration and travel restrictions. Reliability was acceptable (*α* = 0.79) for the immigration scale, and excellent for travel restrictions (*α* = 0.92).

*Racial bias*. To assess racial bias, we included a feelings thermometer and asked participants how warm or cold they felt towards the following ethnic minority groups: Asian Americans, Latinos/Hispanics, Blacks/African Americans, American Indians, Arab Americans, and Muslims. These items reflected *personal* racial bias. However, given evidence for the importance of perceived public stigma on individual behavior [56], through the “bias of the crowds” phenomenon [57], we also asked participants how warm or cold they thought *most Americans* felt towards these groups, as a proxy for individual stigma that is not influenced by social desirability. These items reflected *perceived public* racial bias. Both the personal (*α* = 0.95) and perceived public racial bias (*α* = 0.93) scales had excellent reliability. Lastly, we asked a single question from Craig and Richeson assessing the extent to which participants agreed or disagreed with the following statement: “As the status of racial minorities improves, the status of White Americans goes down” on a 7-point Likert scale, to assess perceived group-status threat [12].

*COVID-related financial impact*. Participants were asked the yes/no question, “Have you or any member of your household recently lost your job or significant source of income as a result of the COVID-19 (coronavirus) outbreak?” to assess for financial impacts of the pandemic [21].

### Results

#### Statistical analyses

Similar to Study 1, ANOVAs tested for differences in participant demographics by condition. ANOVAs also assessed for differences by political party (i.e., Republican, Democrat, Independent) in our primary outcomes. Finally, t-tests with Bonferroni corrections assessed for differences in the endorsement of travel restriction by nationality.

For preliminary analyses, one-way ANCOVAs were again used to assess for condition effects on outcome. LSD tests revealed the directionality of effects for significant findings. Unlike Study 1, we investigated a potential mediator (i.e., group-status threat) and moderators (e.g., employment status) of condition effects on outcomes. Using the PROCESS macro for SPSS [58], we performed mediation analyses with 5,000 bootstrapped iterations. For all dependent variables (i.e., conservatism, xenophobia, and racism), we tested the effects of condition (i.e., threat vs. control) on perceptions of group-status threat (path *a*), group-status threat on outcomes (path *b*), the direct effect of condition on outcome (path *c*), and the indirect effect of condition on outcome via the mediator of perceived group-status threat (path *ab*). Although all pathways were considered, based on relatively new guidelines by Hayes and other experts, only a significant indirect effect (path *ab*) was interpreted as successful mediation [58, 59]. Finally, we conducted two-way ANCOVAs to test for interactions between condition and political affiliation or COVID-related financial impact on outcome. Again, LSD tests were conducted for significant findings.

#### Preliminary analyses

There were no differences between conditions by age, gender, or political affiliation, *p* > .05, although those in the threat condition were more likely to be White, χ2(2, N = 252) = 5.89, *p* = .05. In addition, there was a significant effect of political affiliation on individual bias towards ethnic minorities, *F*(2, 238) = 4.78, *p* < .01, aggregate bias towards ethnic minorities, *F*(2, 234) = 7.92, *p* < .01, overall conservatism, *F*(2, 238) = 113.08, *p* < .01, COVID-19 specific conservatism, *F*(2, 237) = 51.66, *p* < .01, travel restrictions, *F*(2, 238) = 12.87, *p* < .01, and anti-immigration attitudes, *F*(2, 238) = 57.94, *p* < .01. LSD post-hoc tests revealed that Republicans were significantly higher in all the aforementioned attitudes than Democrats or Independents. Similarly, Independents were significantly more likely to endorse overall conservatism, COVID-19 specific conservatism, travel restrictions, and anti-immigration attitudes than Democrats. However, there were no significant differences between Democrats or Independents in their endorsed personal or perceived public racial bias.

**Fig 3:**
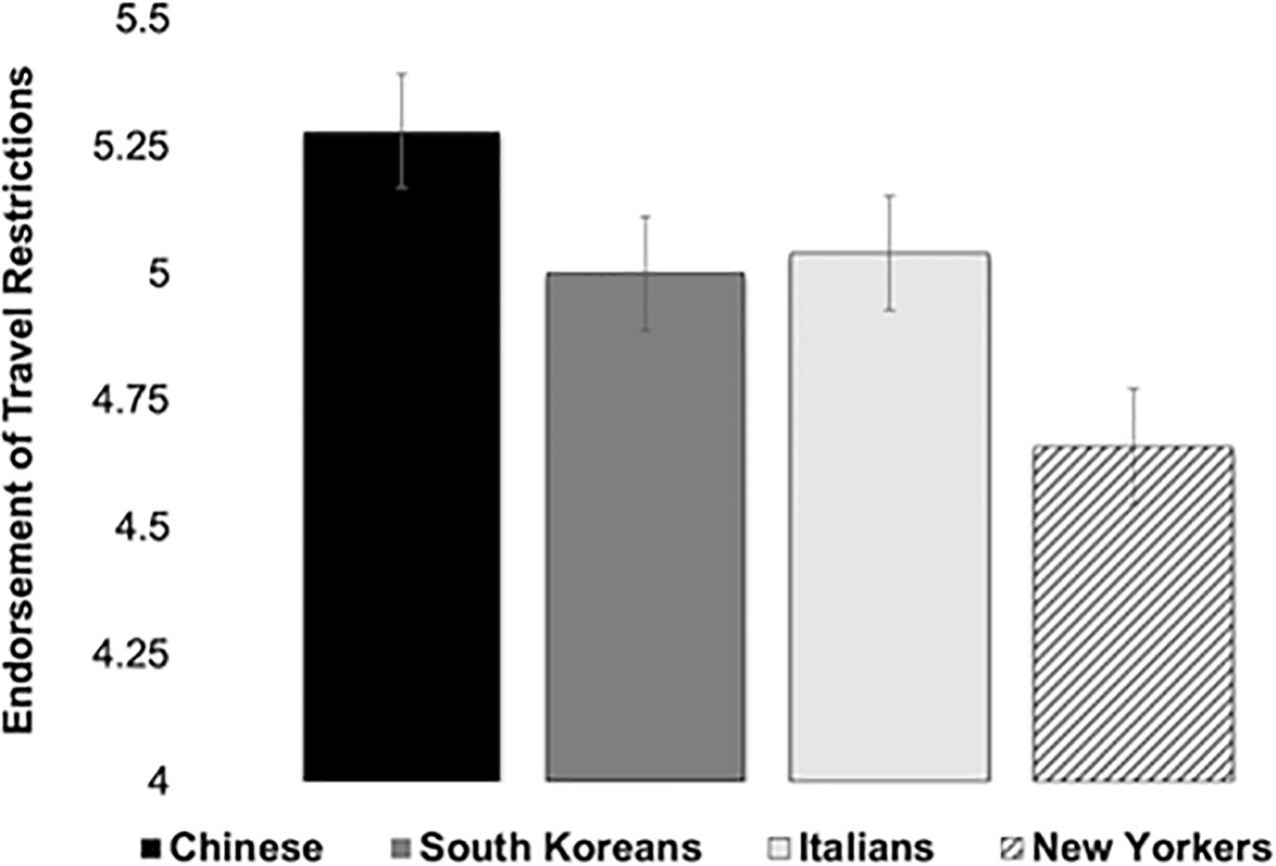
Endorsement of travel restrictions by country in Study 2.

Exploratory analyses found that participants were significantly more likely to endorse travel restrictions against the Chinese than against Italians, *t*(251) = 4.071, *p* < .01, South Koreans, *t*(251) = 5.103, *p* < .01, or New Yorkers, *t*(251) = 5.657, *p* < .01. In addition, they were more likely to endorse travel restrictions against South Koreans, *t*(251) = 3.33, *p* < .01, and Italians than against New Yorkers, *t*(251) = 3.80, *p* < .01, but there were no significant differences between South Koreans and Italians, *p* > .05 (Fig 3).

#### Primary analyses

**Fig 4:**
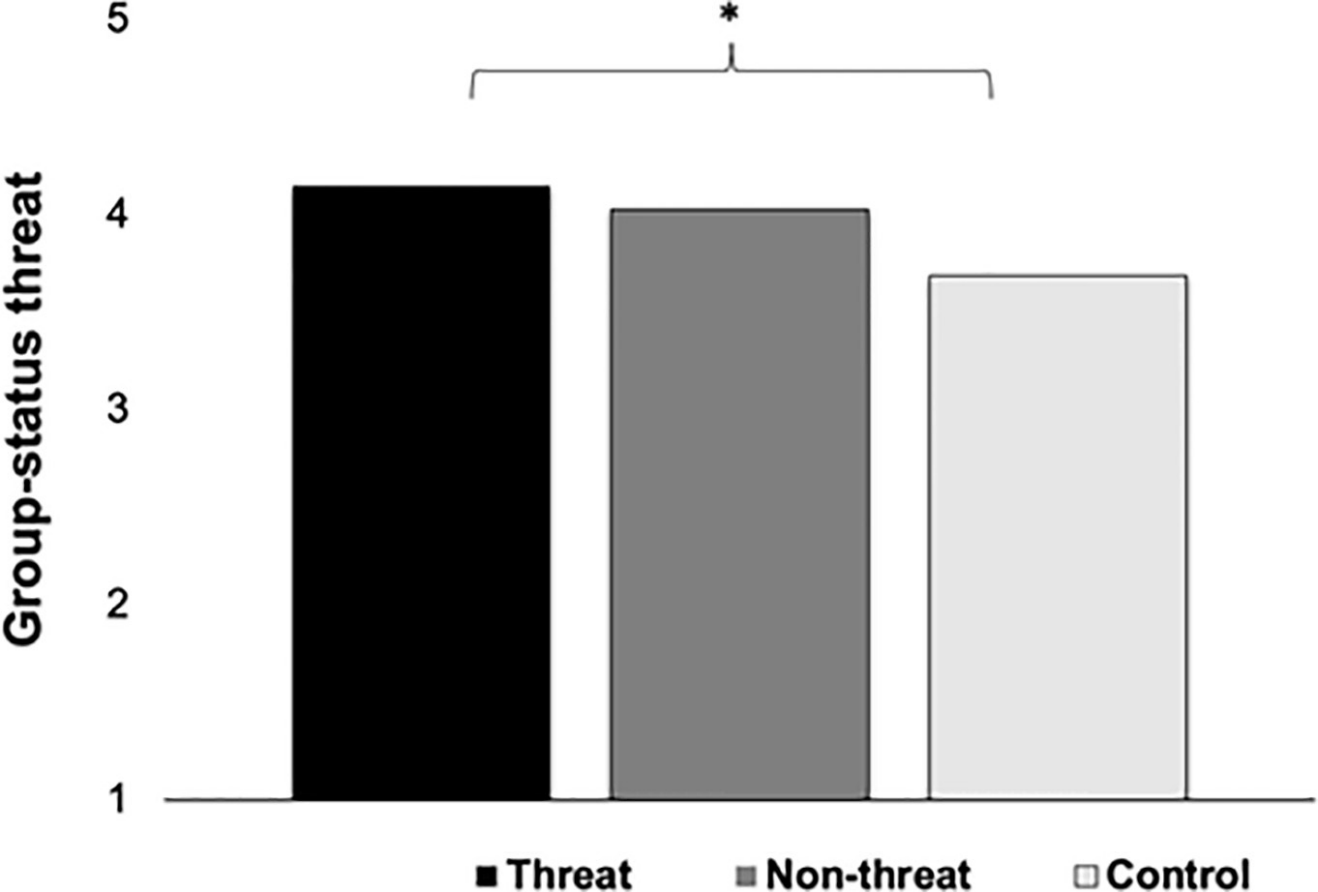
Condition effects on perceived group-status threat in Study 2.

We assessed effects of COVID-19 related threat on conservatism, xenophobia, and racism using ANCOVAs, with age, race, and gender added as covariates, as in Study 1. Consistent with Study 1, the economic threat prime had no effect on overall conservatism, *F*(2, 251) = .43, *p* = .65, η_*p*_^2^ < .01; nor was there any effect on composite attitudes toward COVID-19 policies. However, there was a significant condition effect on perceived group-status threat, *F*(2, 251) = 3.50, *p* = .03, η_*p*_^2^ = .03. LSD post-hoc tests revealed that the economic prime (*M* = 4.20, *SE* = .21) led to significantly greater perceptions of group-status threat than control (*M* = 3.60, *SE* = .21), but the difference between economic threat and non-threat (*M* = 3.74, *SE* = .20) was not significant (Fig 4). There were no direct COVID-19 threat effects on attitudes towards immigration, *F*(2, 251) = .35, *p* = .70, η_*p*_^2^ < .01, composite travel restrictions, *F*(2, 251) = .25, *p* = .78, η_*p*_^2^ < .01 or travel restrictions against any specific group, *ps* > .05, personal racial bias, *F*(2, 251) = .04, *p* = .96, η_*p*_^2^ < .01, or perceived public racial bias, *F*(2, 251) = .42, *p* = .66, η_*p*_^2^ < .01.

**Fig 5:**
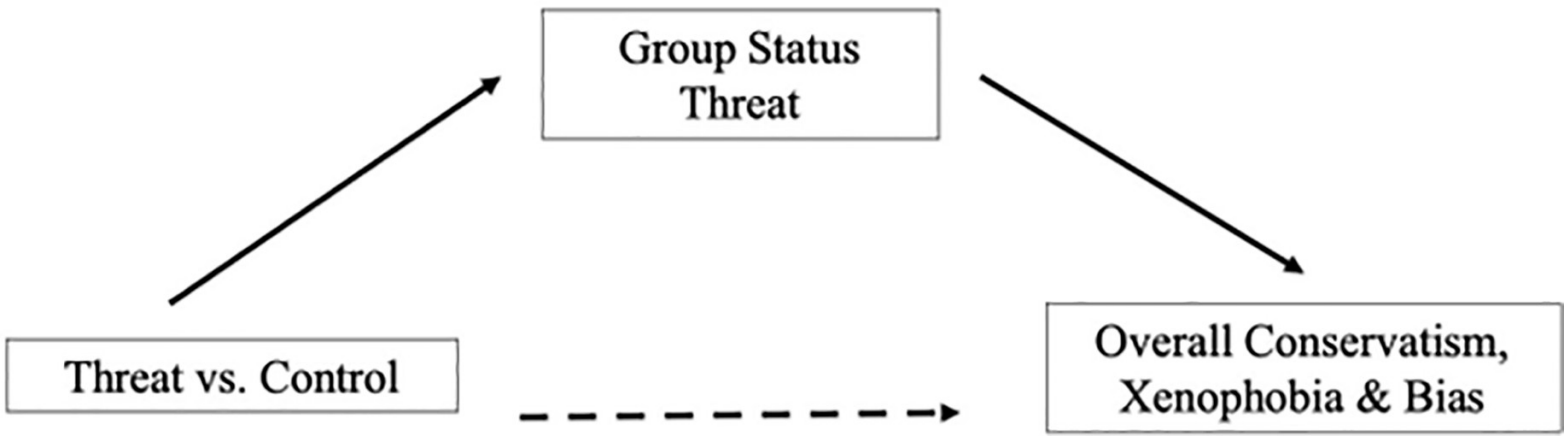
Model of perceived group-status threat mediating condition effects on outcomes.

Next, we assessed perceived group-status threat as a mediator of the effects of threat priming on conservatism, xenophobia, and racism. Because post-hoc tests revealed a significant difference between the economic threat and control conditions only, we removed the non-threat condition from all mediation analyses. Although the direct effect of condition on outcome (path *c*) was not significant for any variables, the indirect effect (path *ab*) [57] was significant for overall conservatism, a*b = 1.56, *SE* = .70, CI_95%_ = [.27, 3.03], immigrant attitudes, a*b = .49, *SE* = .23, CI_95%_ = [.10, .99], personal racial bias, a*b = 1.06, *SE* = .59, CI_95%_ = [.13, 2.41], composite travel restrictions, a*b = .77, *SE* = .39, CI_95%_ = [.10, 1.64], as well as travel restrictions against Chinese nationals, a*b = .19, *SE* = .10, CI_95%_ = [.03, .41], South Korean nationals, a*b = .21, *SE* = .11, CI_95%_ = [.02, .44], and Italian nationals, a*b = .20, *SE* = .10, CI_95%_ = [.02, .42] (Fig 5). These data indicate that the economic prime indirectly predicted increased conservatism and bias through the mechanism of perceived group-status threat.

**Fig 6:**
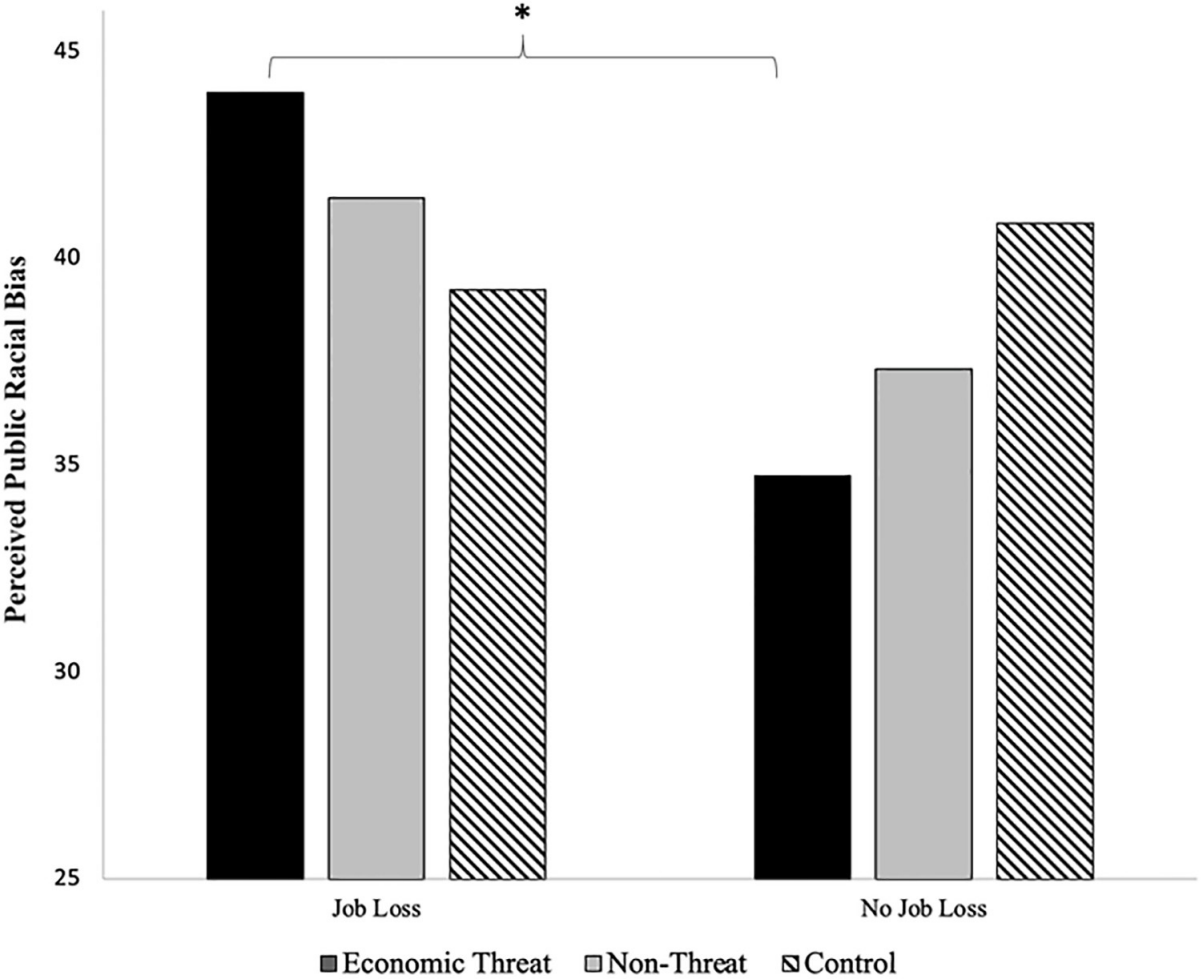
Interaction between condition and financial impact of COVID-19 on perceived public racial bias in Study 2.

Finally, we investigated political affiliation and the financial impact of COVID-19 as potential moderators of effects. No significant interactions between condition and political affiliation emerged. However, COVID-related financial impact significantly moderated condition effects on perceived public racial bias, *F*(2, 234) = 3.38, *p* = .04, η_*p*_^2^ = .03. LSD post-hoc testing revealed that for those who had lost income due to COVID-19, economic threat led to significantly higher perceived public racial bias (*M* = 43.44, *SE* = 2.08) than non-threat (*M* = 39.32, *SE* = 2.76) or control (*M* = 40.00, *SE* = 2.19); for those who had not lost income, there were no condition effects (Fig 6). No moderator effects emerged for personal racial bias or any other outcome measures.

### Discussion

Study 2 expanded on Study 1’s findings by assessing the effects of economic threat on racial bias. Our results provide evidence that priming economic threat (vs. non-threat or control) led to greater perceived group-status threat. In addition, we found that priming threat led to greater perceived public racial bias among those who had experienced COVID-19 related financial impact, but not for those who did not lose significant income sources. Contrary to expectations, the main effects found in Study 1 did not replicate in Study 2; priming economic threat did not directly lead to shifts in xenophobic attitudes. However, priming economic threat (vs. control) indirectly increased xenophobia, conservatism, and racial bias through the mechanism of perceived group-status threat, suggesting that the prime might only be effective when the individual’s feelings of perceived threat were made salient. Implications for our findings are discussed below.

## General discussion

The negative consequences of the COVID-19 pandemic, such as over a million deaths and a record high unemployment rate in the United States alone, have been well-documented [19]. Less apparent are the secondary effects of the pandemic on American political attitudes and racial bias. According to the uncertainty-threat model of conservatism, threat can cause conservative shifts in political ideology [8], it remains unclear how COVID-19 as an unprecedented threat may affect conservatism in the United States. The present set of experiments thus investigated the effects of COVID-19-related threats on political conservatism, xenophobia, and racial attitudes. Although prior studies have found COVID-19-related threat to be associated with conservative and xenophobic attitudes in Europe [41, 60], to our knowledge, this is one of the first studies to examine the effects of COVID-19 threat under the uncertainty-threat model of conservatism in the United States.

Study 1 tested two different forms of COVID-19 related threat: economic vs. health consequences. We found that only the economic threat primed a shift in xenophobia; participants who read an article about the surge in COVID-related unemployment were more likely to endorse travel restrictions against South Korean nationals, despite their well-controlled rates of COVID-19 during the time [48]. Contrary to expectations, COVID-19 related health threat had no effect on political attitudes or xenophobia. In the face of the greatest economic recession since the Great Depression, it is perhaps unsurprising that the economic threat might have been more salient for participants than health threats, when only about 0.2% of Americans had contracted the virus at the time of data collection (i.e., April 30-May 4, 2020) [19].

Study 2 then tested the effects of economic threat on racial bias, and investigated potential mediators and moderators of effects. As expected, economic threat led to elevated perceptions of group-status threat, or perceptions of threat to the high status of in-group members by out-group members. Although there were no other direct condition effects on xenophobia and racist attitudes, COVID-related financial impact moderated effects on perceived public racial bias, such that those who lost significant income because of the pandemic had greater racial bias when primed by economic threat than those who were not financially impacted. This is consistent with previous studies demonstrating that that job insecurity was associated with increased conservatism and endorsement of antiegalitarian attitudes [10]. In addition, Study 2 found perceived group-status threat to be a significant mediator of condition effects on outcome, such that the economic threat condition (vs. control) led to greater perceived group-status threat, which then led to increased conservatism, xenophobia, and racial bias.

Overall, results of the two studies are consistent with prior literature on the uncertainty-threat model, and have implications suggesting that COVID-19, similar to other salient threats, may cause conservative shifts. Common to both studies were increased xenophobic and racist attitudes following the economic threat prime. Although there was no mention of China or other foreign countries in the articles participants read, those in the economic threat condition still endorsed travel restrictions against foreign nationals and anti-minority attitudes more than those in other conditions. Under the framework of uncertainty-threat model of conservatism [1, 2, 4], exposure to the threat of COVID-related financial instability in our study may have caused anxiety or uncertainty that was alleviated by anti-immigrant or anti-minority attitudes. Constant threatening news reports and information on COVID-19 [23, 24] may therefore explain, in part, the increased racial violence against Asian Americans across the country. Whether media reports explicitly or implicitly link COVID-19 to China, increased attention on COVID-19 led to increases in racism, and Sinophobia in particular, since the start of the pandemic [27, 29]. Of note, our threat prime increased xenophobic attitudes towards South Koreans, despite their controlled rates of COVID-19 and lack of connection to China. This finding suggests that COVID-19 threat not only increased anti-Chinese bias, but generalized bias against Asians overall.

The present research also has implications for understanding the turbulent 2020 Presidential and Senate elections in the United States. Our study found that COVID-19 economic threat led to increased xenophobia, and indirectly led to increased conservatism, supporting the uncertainty-threat model of conservatism. Indeed, many of the conservative policies typically endorsed by the Republican party, such as greater restrictions on immigration, have been more widely endorsed as a reaction to the pandemic [61]. This may partially explain why the expected “landslide” victory for Joe Biden, the Democratic candidate, did not occur in the 2020 presidential election [62, 63]; instead, Donald Trump won the second highest number of votes in American history, and 47% of the total votes. In general, the results of our study may also have implications for informing the way media and politicians frame threats to the American populace. For example, less emphasis on the Chinese origins of the COVID-19 pandemic, and avoidance of racially insensitive terms (e.g., referring to COVID-19 as “kung flu” [64]) may have lessened the severe anti-Asian bias and violence across the United States in 2020.

It is unclear why the health threat condition had no effect on conservatism. Because the study was conducted in late April/early May of 2020, it is possible that participants had been oversaturated by media coverage of COVID-19 health risks without having experienced them personally, and the article did not effectively prime threat. Although health-related consequences of COVID-19 can be more severe than economic consequences, such as mortality, only 0.2% of Americans had contracted COVID-19 during the time of this study [19]. In contrast, nearly 40% of participants reported experiencing personal economic consequences of the pandemic, so the experimental manipulation may have only been successful in priming financial stress. Another explanation is that economic concerns may have been more salient than health concerns during that time period due to media coverage. For example, the platforms of Democratic candidates in the 2020 elections focused primarily on advocating for policies that would alleviate economic burden, a strategy that experts contribute to their eventual success [65, 66]. Similarly, a recent study found that framing COVID-19 information to emphasize economic risks primed outgroup bias, whereas health risk information had no effects [67]. Future studies should more thoroughly investigate differences between health and economic threats in different contexts.

It is also unclear why economic threat had no direct effect on conservatism in either study. In contrast to prior studies demonstrating that various forms of threat (e.g., job insecurity, mortality, rapid Hispanic population growth) led to increased political conservatism [8, 10, 12], our study found that COVID-19 related economic threat only increased conservatism through the mechanism of perceived group-status threat. One possibility is that threat may have had differential effects on the endorsement of economic vs. social policies. For example, some research finds differences between economic and social forms of conservatism, suggesting that only social conservatism may be protective [68–73]. It is possible that socially conservative but economically liberal policies may have eased COVID-19 related fears. Unfortunately, we did not use established scales that differentiated between the two forms of conservatism [73], and were therefore only able to assess priming effects on overall conservatism. Another possibility is that COVID-19 threat may only have indirect effects on conservatism via relevant mechanisms. In support of this theory, another COVID-19 threat study conducted in the same approximate time frame as ours (e.g., spring 2020) also failed to find direct effects of threat on conservatism, and only found indirect effects through the mechanism of anxiety [41]. Although we found that perceived group-status threat similarly mediated effects, measuring anxiety could have helped clarify our conflicting results.

Our study has several limitations that should be considered when interpreting our findings. First, our relatively small sample of young, predominantly White and male Prolific users limited our ability to perform more nuanced analyses of the data. For example, we were unable to assess whether effects differed between sex, race, and age groups. Second, our study was only conducted in the first few months of the pandemic, and did not consider how reactions to the pandemic rapidly evolved since then. With greater infection and mortality rates, it is plausible that the health threat may have been more salient over time. Third, the directionality of the relationship between our threat prime and mechanism in Study 2 cannot be determined. Based on prior literature [12], it is likely that the prime increased perceived group-status threat, rather than vice-versa, but our data does not allow for us to make this assumption. Fourth, we did not test for other moderators or mediators of effects relevant to the COVID-19 pandemic that were discovered following the completion of our study, such as anxiety or distress [41, 74], nationalism [75], protection efficacy [76], COVID-19 knowledge [77], trust in scientists [78, 79], and trust in the government [43]. Finally, we did not include a manipulation check to assess participants’ feelings of uncertainty, which would provide information on the effectiveness of our threat prime.

In summary, the current study supports the uncertainty-threat model by providing insights on the effects of COVID-19 threat on American shifts in political ideology and bias during the start of the pandemic. It is important to highlight that our study did not find significant differences in the effects of threat on political attitudinal shifts between members of different political affiliations, suggesting that threat may increase conservatism and bias similarly among both conservatives *and* liberals. Similar to other major crises in history of the United States, such as the Great Recession of 2007–2009, and the 9/11 terrorist attacks [5, 6], we found that COVID-19 threat led to increased xenophobia, racial bias, and shifts in political conservatism across diverse populations. The similarities to the 9/11 attacks are particularly pronounced, with the anti-Muslim bias and racial violence in the early 2000s closely mirroring the current surges in Sinophobia during the COVID-19 era. The results of our studies, which demonstrate causal pathways between threat and conservatism or bias, therefore have implications for predicting attitudinal shifts among with the American general public in future crises. More research is now needed on the long-lasting effects of the pandemic on the political or social attitudes of the general public, and how this may influence public policy.

## Supporting information

S1 Appendix Study 1 experimental stimuli. (DOCX)

S2 Appendix Study 2 experimental stimuli. (DOCX)
