# Supplementary material for: Effects of COVID-19 related economic threat on political conservatism, xenophobia, and racial bias in the United States
Source: PLoS One. 2024 Sep 18;19(9):e0309766. doi: 10.1371/journal.pone.0309766 (PMC11410237; doi:10.1371/journal.pone.0309766)
Supplement: S1 Appendix — (DOCX) [file pone.0309766.s001.docx]

**S1 Appendix**

**Study 1 Experimental Stimuli**

**Economic threat condition article**

**The U.S. Unemployment Rate is Surging**

By: S. Roberts

A new analysis by the U.S. Bureau of Labor Statistics finds that an unprecedented 4.4 million Americans filed for unemployment last week alone. This brings the total of just the past 6 weeks up to 30.3 million—the biggest jump in history.

This translates to a barely believable 23% unemployment rate— in contrast, the unemployment record was a staggering 24.9% during the Great Depression.


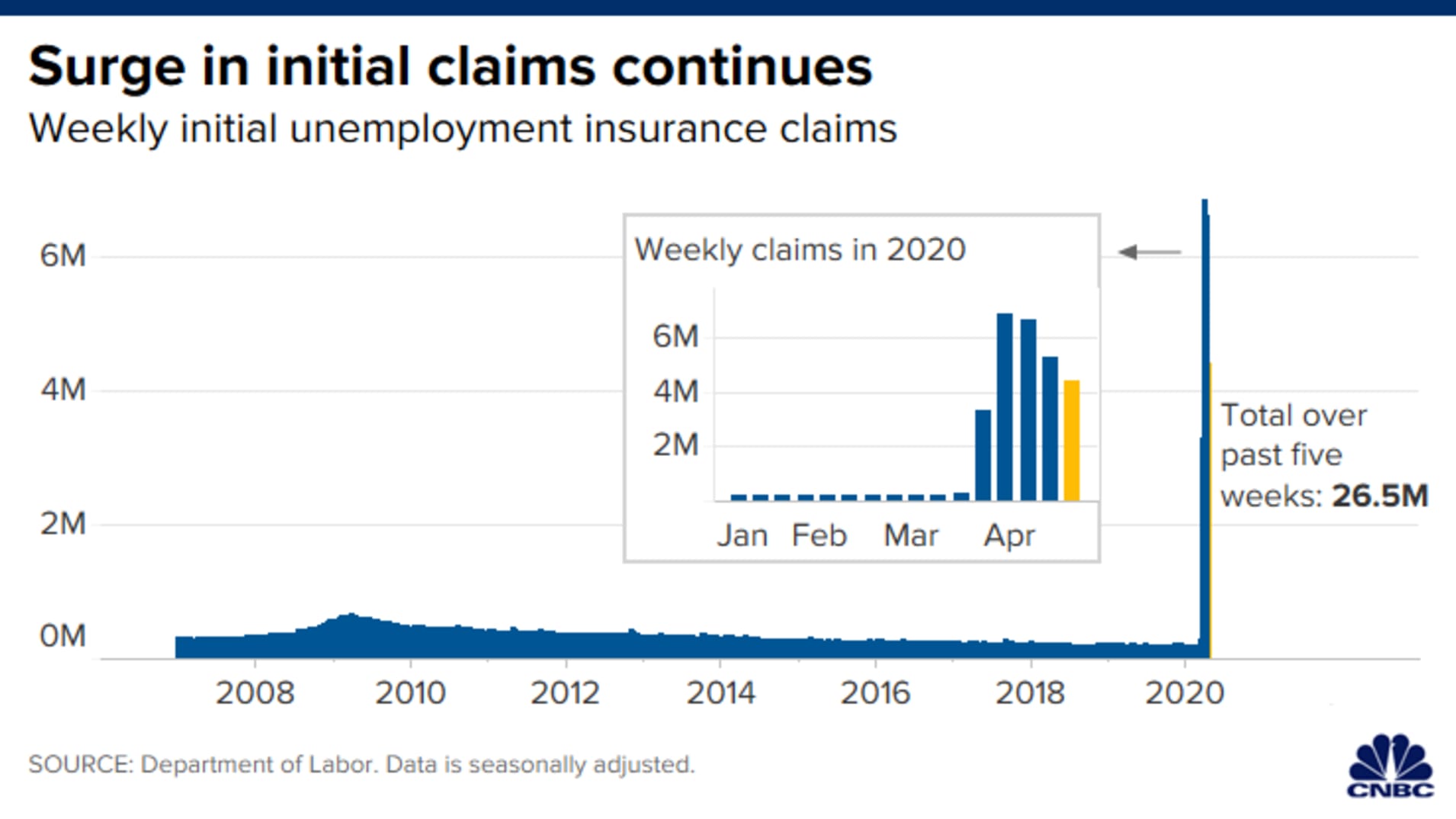
James Knightley, chief international economist at ING, estimates that “less than half of working age Americans will be earning a wage in the next month.” And there is no guarantee it will stop there.

Jobless claims are expected to stay in the millions for several more weeks, as the impact of the coronavirus cascades through the economy. It all adds up to a worse recession than initially thought, and probably a more difficult recovery once the pandemic subsides.

If initial jobless claims don’t begin leveling off, a 30% unemployment rate moves from “the realm of possibility” to “the most likely forecast,” said Claudia Sahm, a former Federal Reserve economist.

**Health threat condition article**

**The Number of U.S. COVID-19 Cases and Deaths is Surging**

By: S. Roberts

A new analysis by the Center of Disease Control (CDC) finds that an unprecedented 1,031,059 Americans have been diagnosed with coronavirus (COVID-19)—the highest in the world. In fact, the U.S. now has three times as many confirmed cases than any other country across the globe.

Coronavirus has also become the leading cause of death in the United States. Approximately 60,000 Americans have died from the virus as of Saturday, a staggering tenfold increase in the month of April alone. According to data by the National Center for Health, this number is far greater than the number of Americans who have died from heart disease, cancer, and other common conditions.

In the hardest hit state of New York, Governor Andrew Cuomo reported that a barely believable one in five residents tested positive for coronavirus in a new study conducted by the state.

However, in 18 other states and the District of Columbia, the COVID Tracking Project finds that rates of infection haven’t peaked yet, and that the number of new confirmed cases will continue to surge through May.

**Control condition article**

**Social Media Use in the U.S. is Surging**

By: S. Roberts

A new analysis by the Pew Research Center shows that an unprecedented 72% of all adults in the U.S. used some form of social media in 2019. In contrast, only 5% of Americans used social media in 2005, translating to a barely believable 14-fold increase in use over the past decade and a half.

Although young adults were the first users of social media platforms, usage by older adults has increased significantly over the past few years. Approximately 70% of adults between the ages of 50-64 report social media use, and 40% of adults 65 and over.


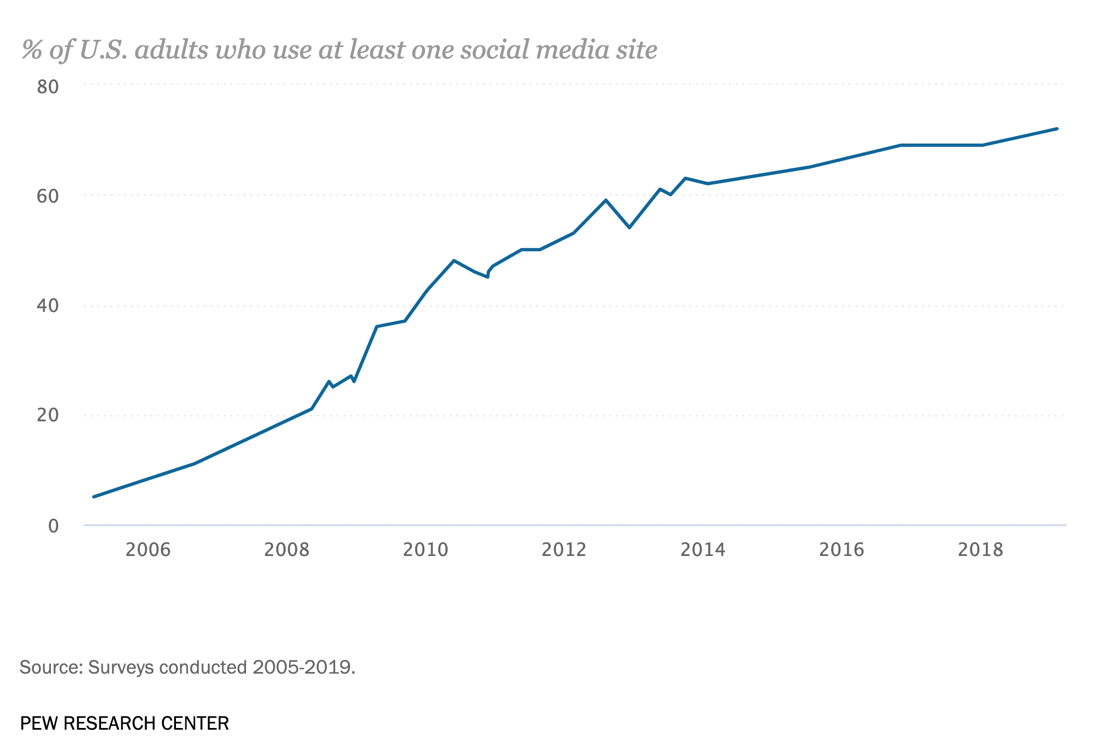


For most users, social media is part of daily life, and research shows that the average American spends 3 hours a day on various platforms. The most used online platforms are YouTube and Facebook, with a staggering 70% of U.S. adults reporting use. Other popular social media platforms include Instagram, Twitter, and Snapchat.

Researchers predict that social media use will continue to surge with the increased access to smartphones and tablets in the United States.
