# Supplementary material for: Effects of COVID-19 related economic threat on political conservatism, xenophobia, and racial bias in the United States
Source: PLoS One. 2024 Sep 18;19(9):e0309766. doi: 10.1371/journal.pone.0309766 (PMC11410237; doi:10.1371/journal.pone.0309766)
Supplement: S2 Appendix — (DOCX) [file pone.0309766.s002.docx]

**S2 Appendix**

**Study 2 Experimental Stimuli**

**Non-threat condition article**

**Zoom Use During COVID-19 Pandemic is Surging**

By: S. Roberts


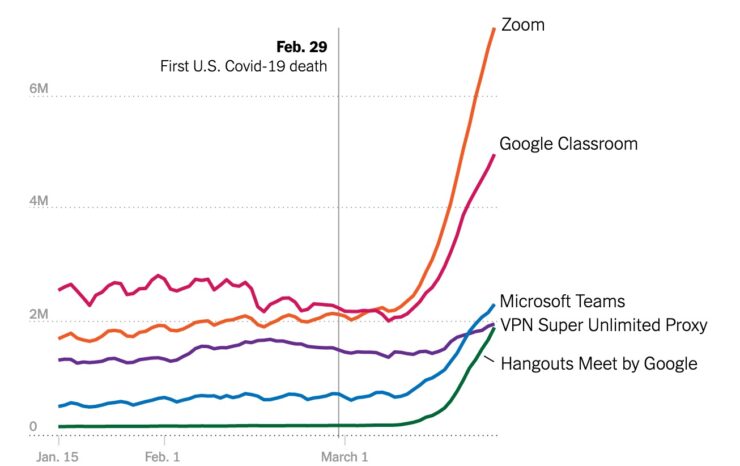
A research report finds that the online meeting platform Zoom has had an unprecedented 30-fold increase in traffic over last 4 months. At the end of December, there were only about 10 million daily Zoom meeting participants. But as of April this year, Zoom had a barely believable 300 million daily users. Since mid-March, Zoom has also become the top free app for iPhones in the United States.

Figure 1 Increased use of video conferencing platforms in the United States

With millions forced to stay home and work and attend classes remotely due to coronavirus concerns, there has been a staggering increase in the use of video conferencing platforms. But none as much as Zoom, which has far surpassed all other platforms as the app of choice for millions across the globe.

The app, which allows users to chat with up to 99 others at the same time, has served a vast variety of functions, such as hosting virtual lectures, church services, doctor’s appointments, and even blind dates.

According to Zoom, “96% of top U.S. universities use Zoom,” and this number is ever increasing during this time of social distancing.
